# Narrative predicts cardiac synchrony in audiences

**DOI:** 10.1038/s41598-024-73066-8

**Published:** 2024-11-02

**Authors:** Hugo Hammond, Michael Armstrong, Graham A. Thomas, Edwin S. Dalmaijer, David R. Bull, Iain D. Gilchrist

**Affiliations:** 1 School of Psychological Science, University of Bristol, Bristol, UK; 2 Bristol Vision Institute, University of Bristol, Bristol, UK; 3 British Broadcasting Corporation (BBC) Research and Development, Salford, UK; 4 Department of Electrical and Electronic Engineering, University of Bristol, Bristol, UK

**Keywords:** Audiences, Immersion, Physiological synchrony, Narrative, Story, Psychology, Human behaviour

## Abstract

Audio-visual media possesses a remarkable ability to synchronise audiences’ neural, behavioural, and physiological responses. This synchronisation is considered to reflect some dimension of collective attention or engagement with the stimulus. But what is it about these stimuli that drives such strong engagement? There are several properties of media stimuli which may lead to synchronous audience response: from low-level audio-visual features, to the story itself. Here, we present a study which separates low-level features from narrative by presenting participants with the same content but in separate modalities. In this way, the presentations shared no low-level features, but participants experienced the same narrative. We show that synchrony in participants’ heart rate can be driven by the narrative information alone. We computed both visual and auditory perceptual saliency for the content and found that narrative was approximately 10 times as predictive of heart rate as low-level saliency, but that low-level audio-visual saliency has a small additive effect towards heart rate. Further, heart rate synchrony was related to a separate cohorts’ continuous ratings of immersion, and that synchrony is likely to be higher at moments of increased narrative importance. Our findings demonstrate that high-level narrative dominates in the alignment of physiology across viewers.

## Introduction

A fundamental aspect of media experiences, be it television, books, live music, movies, theatre, or virtual/mixed reality, is that of immersion. When immersed, the content may easily hold our attention for extended periods during which we lost track of time; fail to respond to other events and people around us; and even temporarily forget to eat, drink, and sleep^2–4^. Immersion within the behavioural sciences has been defined as a ‘state of deep mental involvement in which an individual may experience dissociation from awareness of the physical world’^5^. Cognitive outcomes of immersion include a narrowing of attention towards the media^6^, distorted perception of the passing of time^7,8^, and allocation of cognitive resources to process aspects of the media, including perceptual features, events, and characters^9^. Specific sub-fields including media psychology, neurocinematics^10^, and psychocinematics^11^ have arisen in order to explore these fundamental aspects of cognition during media experiences.

The physical, perceptual, and sensory quality of the media (so-called low-level features) can impact the degree to which we become immersed. In audio-visual media (film and television), immersion is heightened by features that bring the presentation format closer to the limits of the human visual system, such as increased dynamic range^12^, larger screen size^13,14^, or 360-video^15^. Further, perceptual features including motion, luminance, image framing, and shot length can all contribute towards fostering immersion, by guiding eye movements so that audience members look in the same places at the same time, allowing them to follow the higher level story^16–18^. Audience responses are especially synchronous for ‘Hollywood-style’ edited media^10^, where the intention is to direct attention through cinematic and televisual conventions^19^. This attentional synchrony is largely unaffected by viewer’s comprehension of the content, leading it to be termed the ‘tyranny of film’^20,21^. However, it may be broken once motion cues are removed^22^.

Across a range of psychological and media disciplines, a key feature of interest is the story or narrative. For narrative-driven content, the degree to which we become immersed is also thought to be dependent on the representation of high-level abstract properties including characters, events, and story^23^. Cortical activity, as measured using functional MRI (fMRI) appears to signal moments that are emotionally-valent or narratively-important^24^. The temporal boundaries of on-screen events can be predicted from neural activity^25^, which has been used to suggest that individuals segment continuous audio-visual experience into discrete events, to represent, store, and interpret a narrative^26,27^. This neural signature can occur for different stories with similar event schema (e.g. ordering food at a restaurant^28^). This may occur even when viewing remarkably simple narratives, such as a story about rudimentary moving shapes, and these signals can be used to predict an individual’s subsequent interpretation and recollection^29^. Further, evidence from speaker-listener pairs (in which one person describes a scene to a listener) suggests that higher synchrony across speaker-listener fMRI activity^30^ or eye movements^31^ facilitates comprehension of the scene. When eye movement synchrony was increased by manipulating visual cues, comprehension between speaker-listener pairs increased further, indicating a causal relationship between low-level synchrony and high-level narrative coherence^31^.

Under normal circumstances it is plausible that low-level and high-level properties may be correlated. For example, the director of a film may choose to make a particular moment perceptually salient at an important moment in the story, such as a dramatic change in lighting or closer shot scale when the protagonist realises they have been tricked^32,33^. These editing decisions can affect subsequent encoding and recollection of events, for example by foregrounding main characters^34^. A key question then is the extent to which immersion is determined by the physical, sensory, and perceptual properties of the content (so called low-level properties) or by the higher-level cognitive processes as we represent, process, and interpret the narrative.

However, when measuring continuous fluctuations in audience immersion, it can be challenging to separate stimulus-induced changes from individual-level fluctuations and random noise. Given the continuous and complex nature of television and film stimuli, it is difficult to apply the typical experimental approach employed in psychological experiments of constraining additional factors to isolate only the particular variable(s) of interest. One naturalistic approach which circumvents this issue is to measure synchronous audience responses: that is, correlated activity between multiple participants^35^. Audience synchrony for immersive media may exhibit in several signals including fMRI^36^, EEG^37^, secondary-task reaction times^9^, heart rate^38^, skin conductance^39^, and head movements^40^. Audience synchrony can occur when groups watch content together^41,42^ as well as individually. Synchrony may therefore arise via stimulus-features alone, and not necessarily from any interaction between audience members. This temporal synchrony is thought to represent collective attention or immersion^7,43^.

One established signal which synchronises across members is cardiovascular activity. Heart rate synchrony has been demonstrated for both auditory and visual narrative-based content^38^, is especially sensitive to viewer’s attentional and emotional engagement, and can be used as a measure for immersion^44^. Due to dual parasympathetic and sympathetic innervation, heart rate is affected by emotional valence and arousal, as well as attention and effort^45^. Cardiovascular activity is also known to entrain to stimulus frequency and audio-visual rhythms^46,47^, and may therefore be influenced by low-level stimulus properties. As such, heart rate can be moderated by both low-level perceptual and high-level narrative processes. Cardiac monitoring has the added advantages that it is relatively unobtrusive and allows the audience member to engage in the content uninterrupted and in a naturalistic manner, something which is not always possible using other measures such as neural imaging, eye tracking, or continuous response paradigms.

Our study directly investigated the relative contributions of low-level features, and high-level narrative on immersion, as measured using heart rate synchrony. We presented the first episode of a popular television drama to participants in separate modalities (audio or visual). Each modality was time-locked and contained the same narrative information but did not share any low-level features. Assessing synchrony for participants between these groups reveals any effect arising from shared processing of the narrative, whereas synchrony within each group would reflect narrative and low-level properties of that stimulus modality. This design therefore allowed us to determine the relative contribution to the synchronicity from sensory versus narrative factors. Further, we validate the use of heart rate synchrony as a measure of immersion, by comparing synchrony to a separate cohorts’ continuous ratings of immersion.

## Results

In this study, we aimed to better isolate the effects of narrative on viewer immersion. Participants (*N* = 60) were presented with a 55-minute episode of *The Tourist*, a mainstream drama produced by Two Brothers Pictures Limited for the BBC (see Methods for a description), which was available in the UK on the BBC’s on-demand streaming service iPlayer. To isolate the effects of narrative, we presented each participant with one of two conditions: visual or auditory. In the visual-only condition, participants viewed the video track with subtitles (also known as captions) but without sound. In the audio-only condition, participants listened to the audio track which contained the main audio mixed with audio description. The subtitles and audio description were produced for the BBC by Red Bee Media and available as options for this programme on BBC iPlayer. The subtitles convey the spoken words in the programme along with key sound effects^48^ whilst the audio description provides information about the visual content of the programme, albeit restricted to the gaps in the dialogue^49^.

We pre-registered the study including several hypotheses. The first was that narrative information alone would lead to heart-rate synchrony between participants in the audio-only and visual-only condition, significantly greater than zero (H1). Further, we expected that low-level sensory properties over and above the narrative information, will lead to greater synchrony within the audio-only and visual-only conditions, compared to between the conditions (H2). We further predicted that heart rate synchrony would be predictive of self-reported immersion for the audio-only and visual-only conditions (H3). Finally, we predicted that heart rate synchrony over time would relate to how engaging a group of independent raters coded that moment (H4).

### Heart rate synchrony arises from shared processing of the narrative

**Fig. 1:**
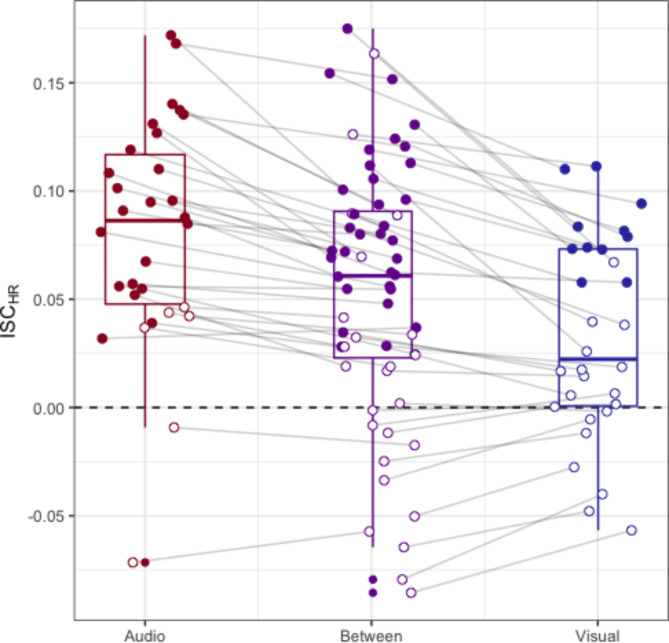
Heart rate intersubject correlations for audio-only, visual-only, and the between-group narrative comparison. Boxplots show medians and interquartile range, and the horizontal dashed line represented zero correlation. Each point shows the average intersubject correlation for one participant. For the audio-only and visual-only conditions, ISCs were calculated for each participant with each other participant in the same group. For the between-group narrative comparison, ISCs were calculated for each participant with participants in the other condition. The grey lines denote the same participant in each condition (within a group, or between-groups). Filled circles denote significance for each participant at *p* < .05, using a circular shuffle technique with 10,000 iterations and a false discovery rate of *q* = 0.05. To calculate significance for each participant, heart rate was shifted circularly by a random value, and ISCs with other participants were recomputed. The proportion of significant ISCs below the true ISC value was used to determine significance.

We capitalised on the complete separation of audio and visuals in our design, by computing heart rate synchrony between audio-only and visual-only conditions and comparing this to heart rate synchrony within each of these conditions. These results are summarised in Fig. 1. This between-condition inter-subject correlation (detailed in the Methods) reflected only shared processing of the narrative (shared between conditions), rather than synchronisation from any low-level visual or auditive features (not shared between conditions).

At the group level, heart rate synchrony for the narrative was significantly different from zero [mean *r* = .054, *t*(59) = 6.99, *d*(95% CI) = 0.90(0.60, 1.20), *p* = 2.81 × 10^−9^]. As a second test, we computed whether individual-level synchrony was different from a circularly shuffled null distribution and found that it was for 31 out of 60 participants (chance level is 3 out of 60). This non-parametric permutation test is resilient to violations in independence of data, which may be broken by computing pairwise correlations between participants. These results suggest that heart rates were more synchronised between individuals than could be expected by chance, and that this synchrony was driven by the isolated effect of narrative. As such, our first pre-registered hypothesis (H1) is supported.

Heart rate synchrony within each condition was also significantly different from zero [audio-only: mean *r* = .081, *t*(29) = 8.53, *d*(95% CI) = 1.56(1.01, 2.09), *p* = 2.16 × 10^−9^, and 24/30 individuals; visual-only: mean *r* = .032, *t*(29) = 3.74, *d*(95% CI) = 0.68(0.28, 1.08), *p* = .0008, and 11/30 individuals]. This contextualises the effect size of narrative synchrony, which is comparable to the effect sizes of within-modality synchrony. This is notable, because it suggests that narrative was the main driver of heart rate synchrony, and that low-level features within each modality had little or no impact. Further, these effect sizes (*r* = .032 − .081) are on a similar scale to previous findings that heart rate synchronises to audio or visual narratives^38,44^. We note that this partially supports our pre-registered hypothesis H2, given that audio-only synchrony appears greater than synchrony arising from the narrative, whereas visual-only synchrony is lower than synchrony arising from narrative. We discuss the implications of these results in the discussion.

### Narrative is much more predictive than low-level salience

It is possible that between-group synchrony is artificially inflated by co-occurrences in perceptually salient visual and auditory features in each modality. For example, higher volume may co-occur with increased brightness and motion, if for example, an explosion is shown on-screen. In this way, correlation between groups would not arise from simply the narrative. To address this possibility, we computed and modelled the effects of low-level visual and auditory features. For the low-level visual features, we used a well-established saliency model^50^ to compute conspicuity and saliency maps. This biologically-inspired model of saliency computes 72 low-level features for colour, intensity, motion, flicker, and orientation, at various scales which simulate different retinal field populations. We computed the root mean square of each conspicuity map (colour, intensity, motion, flicker, orientation) averaged over 1s intervals. For the audio data, we computed root mean square energy as a measure of volume (RMS volume).

To assess co-occurrence between visual and auditory features, we used a multiple linear regression, to predict RMS volume from visual saliency. We found that visual and auditory salience were related [*r*^*2*^ = 0.096, *F*(6, 3345) = 59.43, *p* = 2.2 × 10^−16^].

We then aimed to predict heart rate in each condition, from both low-level features and heart rate of participants in the other condition (as a proxy for narrative), allowing us to determine the relative weightings of each. Table 1 compares three nested multiple regression models: a null model predicting heart rate from only time (in seconds), a narrative model predicting heart rate from heart rate in the other condition, and a low-level model containing each of the previous parameters with the addition of each saliency feature and RMS volume. We did this in each direction: e.g. predicting both visual and audio heart rate. We compared these models using Bayesian Information Criterion (BIC), which estimates model performance through a balance of goodness of fit and penalisation function for the number of parameters. A lower BIC indicates better model performance. As shown in Table 1, the best performing models include both narrative and low-level saliency. However, comparing variance explained by each model, saliency parameters explain 1.7–2.2% variance, compared to 17–23% from narrative, demonstrating that narrative is approximately 10x as predictive.

**Table 1:** Model comparison predicting heart rate (HR) in condition. Bold indicates the best performing model, as identified using bayesian inference Criterion (BIC). RMS represents root mean square energy of the audio track, a measure of volume and auditory salience.

Model	Predictor	df	Parameters	*R* ^2^	BIC
Null	Visual HR	3	Seconds	0.131	332.98
Narrative	Visual HR	4	Seconds + Audio HR	0.360	-684.78
**Narrative +** **Low-level**	**Visual HR**	**10**	**Seconds + Audio HR + RMS + Orientation + Colour + Flicker + Intensity + Motion**	**0.377**	**-736.43**
Null	Audio HR	3	Seconds	0.365	713.28
Narrative	Audio HR	4	Seconds + Visual HR	0.531	-301.07
**Narrative +** **Low-level**	**Audio HR**	**10**	**Seconds + Visual HR + RMS + Orientation + Colour + Flicker + Intensity + Motion**	**0.553**	**-415.70**

### Self-reported retrospective engagement does not align with physiology

We found that heart rate level was higher in the audio-only than the visual-only conditions [*t*(57) = 2.17, *p* = .034, Cohen’s *d*(95% CI) = 0.43(0.08, 0.94)]. Self-reported engagement data was collected from the same participants after viewing using two scales: the Film Immersive Experience Questionnaire^51^ and the Narrative Engagement Scale^3^. Engagement ratings for the Film Immersive Experience Questionnaire^51^ were significantly higher in the visual-only condition [*t*(53.7) = 3.12, *p* = .003, *d*(95% CI) = 0.81(0.28, 1.33)]. Specifically, ratings in the visual condition were significantly higher for subscales indexing Captivation: which measures viewers’ interest, motivation, and enjoyment [*t*(53.8] = 3.35, *p <* .001, *d*(95% CI) = 0.87 (0.33, 1.39)]; and Real-world Dissociation: which assesses viewers’ awareness of their surroundings [*t*(57.5) = 3.15, *p* = .003, *d*(95% CI) = 0.81(0.28, 1.34)]. Given this scale was devised and validated to assess differences in display format (screen size), it is unsurprising that scores in the visual condition were higher. Engagement ratings measured through the Narrative Engagement Scale^3^ did not significantly differ between conditions [*t*(58.0) = 2.00, *p* = .051, *d*(95% CI) = 0.52(0.00, 1.03)]. As this scale assesses individual’s engagement within the story, this suggests the narrative was equally effective at immersing audiences in both conditions. However, ratings of the Attentional Focus subscale, which concerns the viewer’s level of focus or distraction, were significantly higher in the visual condition [*t*(58.0) = 3.52, *p* = .001, *d*(95% CI) = 0.98 (0.37, 1.44)], suggesting audiences experience of distraction and mind-wandering was greater in the audio-only condition.

These results show that self-reported engagement was higher when participants viewed visual events with subtitles (but no sound), even if heart rate was higher for the audio track with audio description (without visuals) for the same events.

These analyses were not included in our pre-registration, however, were conducted to provide some further background to our effects. These results may contextualise other findings such as^52^ who also note discrepancies between self-reported engagement and physiology. The implications of this are discussed further in the discussion. However, we note that as these analyses were not included in the pre-registration, we did not specifically power to detect these effects. A post-hoc power calculation revealed that the effects documented in this section (*d* = 0.52, *n* = 30 per group) had an achieved power of *b* = 0.52. A sensitivity analysis revealed that we had sufficient power here to detect effects of *d* = 0.74 (at *b =* 0.80). As such, results which fall below this effect size are not sufficiently powered and are not informative.

We predicted that heart rate synchrony would be predictive of self-reported immersion for the audio-only and visual-only conditions (H3). However, there was no significant correlation between heart rate ISCs and the narrative engagement scale (*r* = .120, *p* = .360), or heart rate ISCs and the film IEQ (*r* =-.060, *p* = .650). This was surprising as our previous work had shown a strong association^44^. However, in contrast to the current study our previous work used clip durations that were very short (132–252 s). One explanation for this discrepancy is that retrospective questionnaires are a poor measure of engagement for longer duration content because of the limitations of human memory (see^53^ for a similar result and explanation for live performance).

### Heart rate synchrony relates to immersive moments

**Fig. 2:**
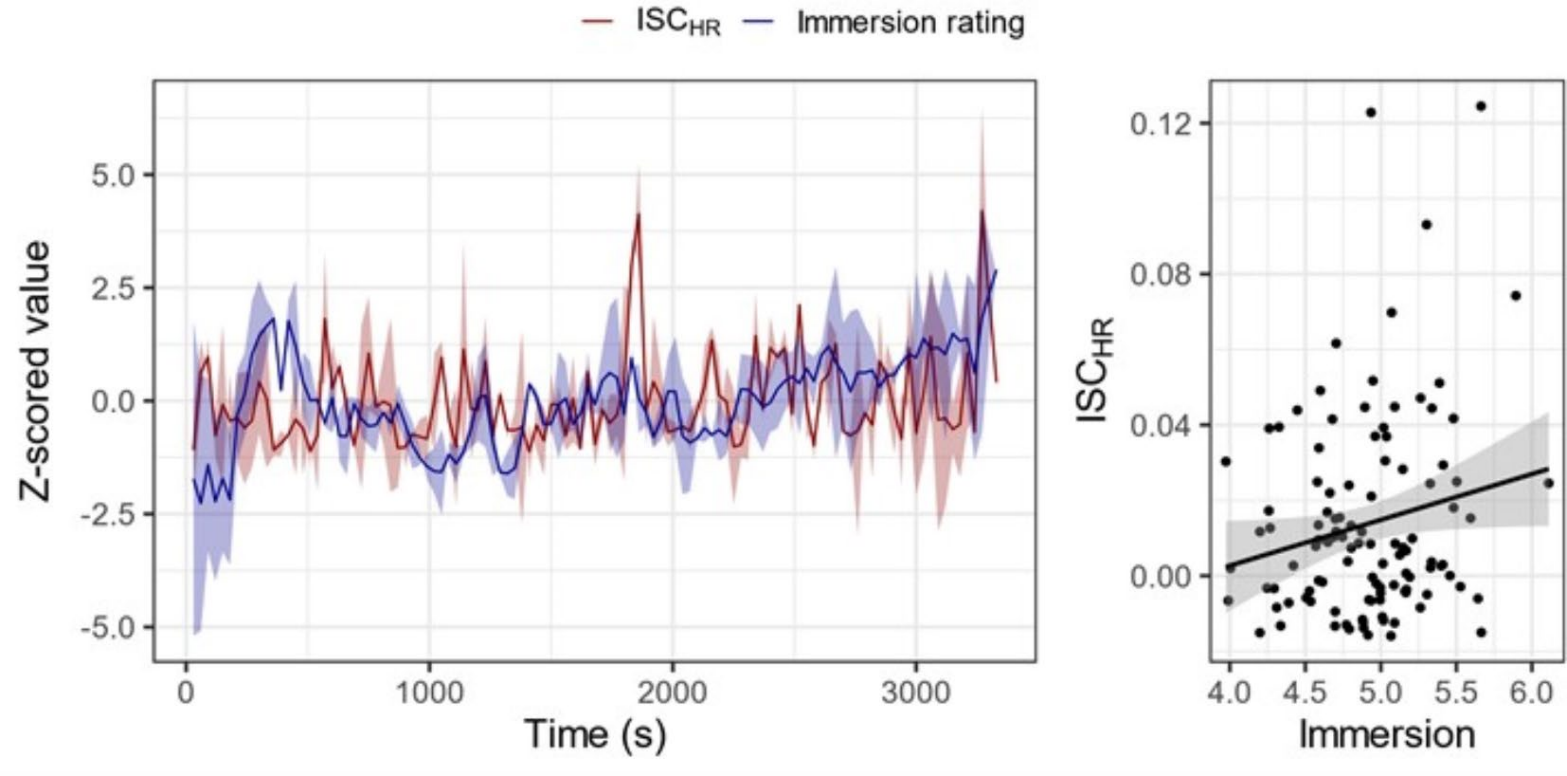
Correlation between heart rate ISC and continuous ratings of immersion. Left: Heart rate ISC and immersion ratings over time, averaged across all participants. Filled area represents 95% CI. Right: Correlation between heart rate ISC and immersion ratings. Each point represents the average rating and ISC at one time point. Filled area represents 95% CI.

A separate group of participants (*N* = 35) viewed the episode of The Tourist with both sound and vision, without subtitles or audio description, while being probed to rate their immersion using a 7-point scale at 30 s intervals. This was conducted only as a secondary validation to confirm that heart rate synchrony is related self-reported immersion, as in previous research^44^, without relying on single retrospective estimates of immersion as in the section above. This section assesses our pre-registered hypothesis H4. Participants’ ratings of immersion were reliable [correlation over time, *r* = .087, *p* = 2.2 × 10^−16^], and related to heart rate synchrony: higher immersion was significantly related to higher heart rate synchrony [*r* = .208, *p* = .029], see Fig. 2. Note that for this analysis, heart rate synchrony was computed on the full pairwise comparisons across all participants, between both the audio-only and visual-only conditions. As seen in Fig. 2, there may be a linear trend of time – where both heart rate synchrony and immersion ratings increase as the content progresses. This effect was accounted for in the previous models in Table 1, by including a linear effect of time.

**Fig. 3:**
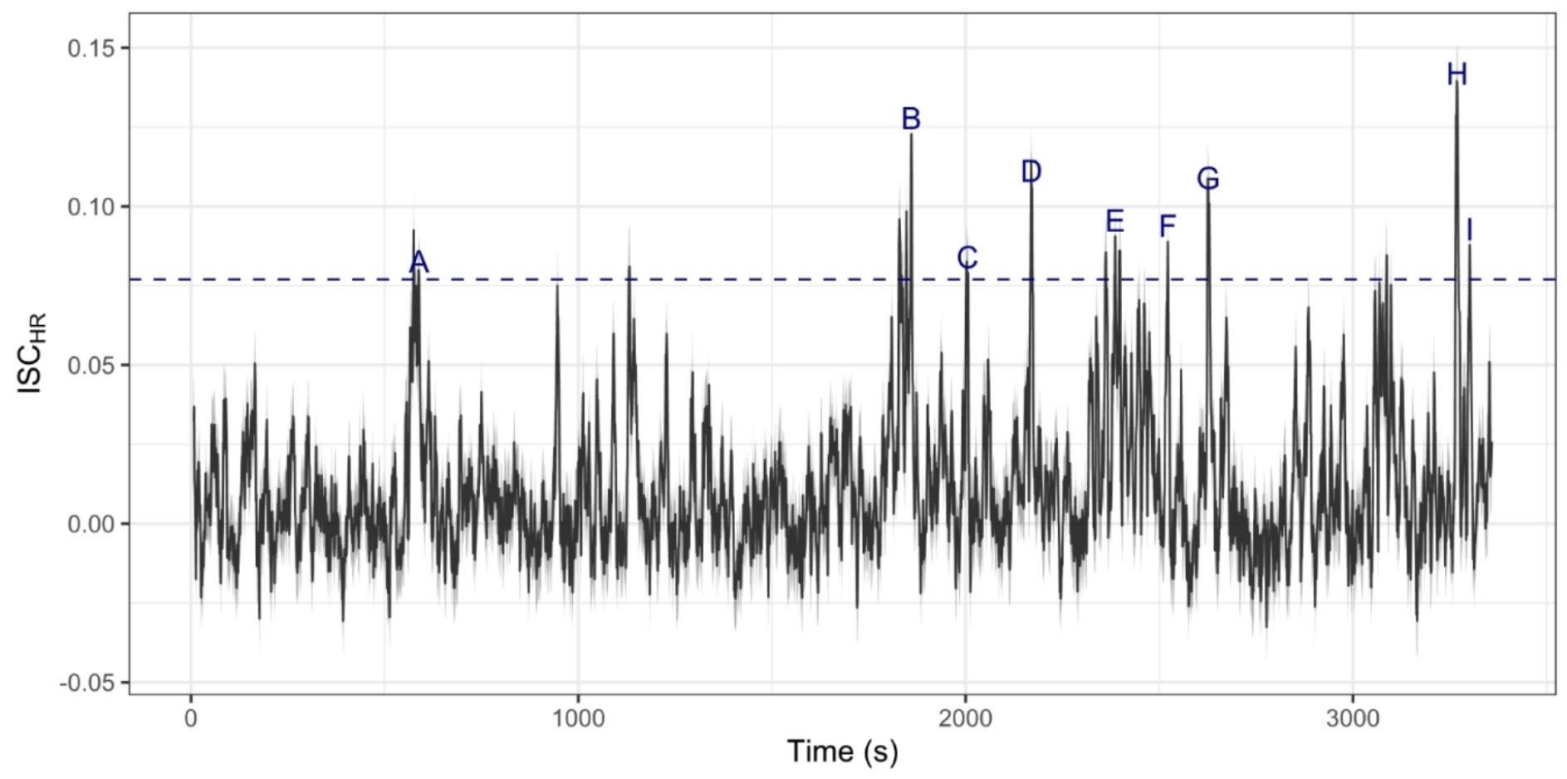
Heart rate ISC across time. Lighted grey area denotes 95% CIs. Dashed line indicates 3 SDs above the mean. Labels depict events, described below. Exploratory investigation of these events, shown by the labels and descriptions, may suggest these points relate to moments of narrative importance or emotional saliency. A. The man is placed in an MRI scanner, struggling to recall aspects of his life. He asks for a song to be played, but then cannot recall it, as it becomes clear that he has amnesia from the car crash. B. The man and Luci, a waitress, walk out of the restaurant. The restaurant explodes behind them. C. The man and Luci speculate that someone may be trying to kill him. D. The man arrives at a bed and breakfast, with insufficient money to pay for a room. The sympathetic owner lets him stay anyway. E. The man and Helen, a policewoman, are talking on the phone. Helen reveals the police found a camera in the wreckage of his car, which may provide information on his identity. They also share a joke which came up earlier. F. The cowboy arrives at the hospital, and asks to see the man. G. The cowboy leaves the hospital, putting on his hat and whistling a tune heard as he earlier attempted to kill the man. It becomes clear he is the truck driver who originally attempted to kill the man. H. Luci, the waitress, is at home scrolling through her phone. She is deleting photos or her and the man together, revealing that she previously knew him. In the background, the song which the man asked to be played in the MRI scanner is playing. I. The man is in a viscerally described, and visually disgusting bathroom.

 We investigated narrative content where synchrony was highest (over 3 standard deviations above the mean), and found that peaks overlapped with important narrative moments, like when a key plot twist is revealed (Fig. 3).

### Skin conductance and movement do not synchronise across individuals

In this study, we also pre-registered the measurement of skin conductance and movement (measured using an accelerometer). These findings are summarised below.

Skin conductance was first z-scored to standardise across participants. Using Welch’s two-sample t-tests, we found no significant difference in mean skin conductance level between the audio and visual conditions [*t*(38.2) = -0.21, *p* = .833]. Next, we calculated skin conductance synchrony for each condition using the same ISC methodology as described in the heart rate analysis. Skin conductance ISCs were not greater than zero in any conditions: audio [mean *r* = − .002, t(29) = -2.38, *p* = .02], visual [mean *r* = -4.34 × 10^−5^, t(29) = 3.74, *p* = .0008], and narrative [mean *r* = -9.28 × 10^−5^, t(59) = -0.18, *p* = .85]. As a second test, we determined significance for each participant by re-computing ISCs from a circularly shuffled null distribution (described in the heart rate analyses). At an individual level, skin conductance synchrony was significant for 1/30 participants in the audio condition, 1/30 in the visual condition, and 1/60 in the narrative condition (chance level would be 3/60). As such, we can conclude that skin conductance does not meaningfully synchronise across participants in any condition.

Movement was derived from the accelerometer data and was derived as the sum of acceleration in the X, Y, and Z axes. These data are presented in units of *g*, where 1*g* = 9.81 m/s^2^. This was z-scored to standardise across participants. Due to an issue arising with the accelerometer, movement data is not available for the first 10 participants, leaving *n* = 50 for these analyses. Using Welch’s two-sample t-tests, we found no significant difference in average movement across conditions [*t*(24.23) = 1.08, *p* = .291]. Next, we calculated movement synchrony for each condition using the same ISC methodology as described in the heart rate analysis. Movement ISCs were not greater than zero in any conditions: audio [mean *r* = − .0005, t(23) = -2.38, *p* = .730], visual [mean *r* = 5.14 × 10^−5^, t(49) = 3.74, *p* = .965], and narrative [mean *r* = -9.28 × 10^−5^, t(59) = -0.18, *p* = .955]. As a second test, we determined significance for each participant by re-computing ISCs from a circularly shuffled null distribution (as in the heart rate analyses). At an individual level, movement synchrony was significant for 1/24 participants in the audio condition, 0/26 in the visual condition, and 1/50 in the narrative condition. As such, we can also conclude that audience movement does not synchronise across participants in any condition.

## Discussion

Recent evidence has demonstrated that heart rate synchronises across individuals when engaging with audio-visual media^38^. Here, we show this synchronisation can occur in response to narrative information alone: i.e. between the same content presented in separate modalities. We find that narrative is a substantially better predictor of heart rate across individuals than low-level visual or auditory salience, suggesting this physiological moderation reflects aspects of higher-level amodal cognition. Finally, we show this synchrony relates to continuous fluctuations in audience immersion.

When perceiving the world, we segment continuous perceptual input into discrete events, centred around details such as characters, character interactions, spatial locations, and temporality^54^. Several studies suggest that when consuming media, we use the same mechanisms as in real life to construct event representations^55^ and interpret characters’ emotional states^56^. To this extent, fiction has been considered a simulation of social worlds^23,57^. One interpretation of our results is that cardiac synchrony between audience members reflects generation, maintenance, and updating of these mental representations. While we do not know which component(s) of narrative drives cardiac synchrony in our study, exploratory investigation shown in Fig. 3 suggest synchrony is higher during moments which are cognitively demanding (e.g. a twist is revealed) or emotionally-salient (e.g. evoking empathy with a character). These narratively-important moments may narrow attention^6,58^, reflected here through temporal alignment of audience physiology.

We did not find a strong exogenous effect of audio-visual features on heart rate synchrony, in contrast to the ‘tyranny of film’ hypothesis that these low-level features such as motion drive attention. This may suggest these low-level visual features direct attention to facilitate event-representation and comprehension^59^, but are not necessarily represented in rich detail by themselves. Evidence from change blindness research, that observers can fail to attend to, or notice, prominent changes in the visual environment, would seemingly provide support for this view^60^. Within audio-visual media, a further example is how audience members often fail to notice shot cuts^61^, except when presented at breaks during scenes^55^. However, when the narrative is less strong it may be that the perceptual qualities of the content have an effect on immersion^12^. Another possibility is simply that any effect of low-level features on physiological responses, for example cardiac deceleration often associated with orienting towards novel stimuli, is quick to habituate^62^. However, it is also possible that eye movements and physiological responses are tracking difference processes. The scene perception and event cognition theory^63^ is one model which distinguishes front-end (within a fixation) from back-end processes (over several fixations). It is feasible then that eye movements are driven more by these front-end processes, whereas heart rate synchrony relates more to back-end processes. Previous research has demonstrated that attentional modulations on heart rate synchrony are modulated at a frequency of around 5–10 s^38^, suggesting that this is a slower process than eye movements.

Our study demonstrates a relationship between heart rate synchrony and audiences’ subjective evaluations of immersion over time. Previously, heart rate synchrony has been linked to immersion across genres of content, in particular, emotional and attentional engagement^44^. Immersion is often considered to facilitate emotional processes, with increased immersion shown to increase story-consistent emotions and empathy^64–66^. Models of immersion often include narrative factors such as empathy, suspense, and curiosity^67^. However, the current study does not show a relationship between individual heart rate and self-reported retrospective engagement, in contrast to some previous evidence^52^. We suggest that this may represent a possible distinction between retrospective immersion ratings, and moment-by-moment fluctuations. However, this may also reflect limitations in the statistical power of this analysis (noted in the methods section).

Other work has compared physiological responses in different modalities and linked this to differences in engagement. For example^52^, found greater heart rate levels when listening to audiobooks than films (although this study was unable to assess physiological synchrony as the content was not time-synchronised). Similarly, we demonstrate increased heart rate synchrony when consuming audio-only than visual-only content, and additionally find greater heart rate synchrony for audio than visual content. We suggest this finding reflects differences in the attentional demands of each modality: auditory information was presented in only one stream. In contrast, attending visual information is a more active process^68^: viewers had to make decisions about where to fixate, and balance fixating on-screen events with the subtitles, thus explaining lower synchrony across individuals.

## Future directions

Much of the literature on audience synchrony during media focuses on synchronisation of neural activity (e.g^10^). However, little is known about how physiological signals explored in the current paper may be a cause or consequence of neural activity. Some have suggested that signals with a strong brain-body relationship, including heart rate, are more likely to synchronise across individuals^69,70^. One candidate for this neural underpinning is the default mode network (DMN), which has been implicated in processes including event segmentation, perspective-taking, and sense-making (for a review, see^71^), and is related to narrative information such as twists^27^. DMN activity during cognitive and emotional tasks also co-occurs with respiration and heart rate changes^72^, offering one pathway for cardiac activity to synchronise across individuals.

Behavioural and brain science often argues that humans are visually dominant creatures^73^. However, when viewing dynamic stimuli, changes in perceptual features often go unnoticed^60^. We argue instead that humans are story driven. When viewing media, as in the real-world, we construct event models, and attempt to understand the thoughts and beliefs of others. The current work suggest that it is this process which determines immersion and synchronises physiological responses. However, we note that while the current work demonstrates narrative alone may synchronise heart rate across individuals, we cannot ascertain what features of the narrative drive this. Exploratory investigation from the current work indicates moments of narrative importance or emotional salience would be good directions for future study. We suggest features such as construction of shared mental representations^74^, or emotional evaluations^41^ as potential mechanisms driving heart rate synchrony, which are deserving of future exploration.

## Methods

### Participants

60 participants, largely recruited from the University of Bristol, UK, students and staff population (mean age = 24.0, SD = 7.5, 16 male, 43 female, 1 non-binary) provided informed consent to participate, and were reimbursed with £15 or course credit. Participants had normal-corrected vision, unimpaired hearing, English as a first language (or an equivalent level of fluency), and had not previously seen any of *The Tourist* (BBC, 2022). Ethical approval was granted by the University of Bristol School of Psychological Science Research and Ethics Committee (code: 11235280622), and the study was conducted in accordance with the Declaration of Helsinki.

### Measures

All physiological measures were obtained and aligned using a MP160 (BIOPAC Systems, Inc, Santa Barbara, CA). Synchronisation with the beginning of the content was established using a custom-built interface which detected a 1000 Hz tone inserted at the beginning of the content.

### Heart rate

Electrocardiogram was obtained using the ECG100C module. Electrodes were placed in a Lead-III configuration on participants’ collarbones and lower left rib. Pre-processing of data was conducted in AcqKnowledge (version 5.0.4) software (BIOPAC Systems, Inc). R-R intervals were used to convert ECG into heart rate. Artefacts were identified by hand as periods where R-R intervals were obscured, resulting in an artificially low or high heart rate. These typically co-occurred with higher acceleration signal (e.g. motion artefacts). Artefacts were corrected by taking the mean of the preceding and subsequent value. Data were initially acquired at 2000 Hz and downsampled to 1 Hz following artefact correction.

### Skin conductance

Skin conductance was collected using the EDA100C module, and electrodes were connected to the distal phalanges of participant’s index and middle fingers, on their non-dominant hand.

### Movement

Movement data was obtained using the TSD109C3 triaxial accelerometer module and placed on the wrist of the participant’s non-dominant hand.

### Self-reported engagement

Self-reported engagement was collected via 2 scales. The Narrative Engagement Scale^3^ is a 12-item scale which assesses four dimensions of engagement: Attentional Focus, Emotional Engagement, Narrative Presence, and Narrative Understanding. Each item is measured on a 7-point scale anchored between strongly disagree and strongly agree. The Film Immersive Experience Questionnaire^51^ is a 24-item scale which assesses four dimensions of immersive experience: Captivation, Real-world Dissociation, Comprehension, and Transportation. One question which asks specifically about the graphics and cinematography was excluded from analysis, because it was not applicable to the audio-only condition.

### Stimuli

Participants viewed Series 1, Episode 1 of *The Tourist*. The synopsis for this episode is as follows: “*When a man wakes up in the Australian outback with no memory*,* he must use the few clues he has to discover his identity before his past catches up with him.”The Tourist* was chosen for its appeal as a mainstream drama (the first episode received 6.1 million streams on BBC iPlayer). The episode follows a traditional story arc, and contains dramatic content and mystery at both a sensory-level (e.g. car crash, explosions) and a narrative-level (e.g. the protagonist attempting to discover who he is, and avoid being hunted by an antagonist). We used the full 55-minute episode as it would not be possible to study high-level processes such as immersion without the use of long-form content. Further, we selected the first episode as this doesn’t rely on any previous context.

Participants viewed either a subtitled version with the audio track removed (n = 30), or an audio-described version with the visual track removed (n = 30). We refer to these as visual-only and audio-only conditions, respectively. In the visual-only condition, participants viewed 1920 × 1080p (HD) content. Participants watched alone on an LG OLED Z2 88” television [width = 198 cm, height = 111 cm] from a distance of 160 cm, in a comfortable model living room setup. In the audio-only condition, audio-description was the default enabled on BBC iPlayer. Audio description is typically used by viewers with visual impairments, and describes the events occurring on-screen. Participants listened to the audio using the display’s internal speakers. The screen in the audio condition was set to a neutral grey.

We chose not to include a reference ‘normal viewing’ condition (i.e. including both audio and visual) because a condition that includes a mixture of audio and visual information does not contribute to directly testing our hypothesis. In addition, it is unclear what constitutes ‘normal viewing’ for television content. The use of subtitles may depend on ambient environmental noise levels or partial hearing impairment and recent data suggests that subtitle use may be ubiquitous and widespread in the participants under 30-year-old^75^. There are probably a wide range of ‘normal viewing’ conditions.

### Intersubject correlation

We chose inter-subject correlation (ISC) to assess differences in audience physiology, as by comparing synchronous responses across multiple individuals, this approach discounts intrinsic fluctuations in physiology due to task-unrelated noise, and maximises the signal arising from stimulus-specific features^76^. ISC of physiological signals were calculated by producing a matrix of pairwise correlations between each participant. Each correlation was conducted across time using a 15s rolling window, shifted at 1s intervals. For analyses at a participant level, we then took the median of each row to calculate the ISC for each participant (in this context, the median is considered more accurate and less skewed than taking the mean^77^). For analyses over time, we took the median correlation for each second rolling window, across all participants. It is important to consider that while ISCs calculated in this way may appear low, each ISC is calculated from correlations between individual physiological values and not averages, as is common in much of psychology.

Statistical significance was assessed in two ways. The first was to compare the ISC distribution for each participant to zero, using a one-tailed t-test. This approach provides an easily interpretable way of assessing whether participants were significantly synchronised. A post-hoc sensitivity analysis conducted using G*Power revealed that we have sufficient power to detect an effect of *d* = 0.11 at 95% power, using a sample size of 870 (30 participants x 30 participants – 30 given that each participant’s correlation with themselves will be 1). Statistical significance of individual participant ISCs were calculated using a circular shuffle approach (see^38^). Here, the signal of interest is shifted in time 10,000 times for each participant, and the correlation matrix recomputed. In this way, we create a null distribution which shares the same autoregressive structure as the data. The proportion of correlations in null distribution below the true correlation is then used to compute statistical significance, which is corrected using a false discovery rate of *q* = 0.05. This non-parametric approach was selected as an additional robust test of significance, which accounts for the dependence between pairwise correlation values. We have included both tests in this article to appeal to both readers who seek an easily interpretable metric of synchrony, and readers who wish to see further robust testing.

### Continuous ratings of immersion

50 participants who had not taken part in the main experiment, completed a continuous rating paradigm of their immersion (our target sample size was 30, but we over-recruited to 50, noting the substantially higher attrition rates during online data collection). Our exclusion criteria was the same as the main experiment. Participants viewed the unaltered audio-visual episode of The Tourist, with a 7-point scale visible below the video. Every 30 s, a 1 s 1000 Hz sine wave played, which upon hearing, participants rated their agreement with the statement (*‘I feel very immersed’*). Participants completed the task in a quiet undisturbed location using their own laptop or desktop computer. The experiment was built using PsychoPy 2022.2.4 and hosted online using Pavlovia. Responses with a reaction time over 5 s were excluded, and participants with over 20% missing data overall were excluded from analysis, leaving a final sample of 35 participants (mean age = 19.5, SD = 0.78, 6 male, 29 female).

### Significance statement

The consumption of audio-visual content is a world-wide activity which for many engages many hours a day. For example, in the USA, it is estimated that individuals spent 4 h 42 min each day consuming broadcast or streamed television content^1^. Despite the prevalence of media in our daily lives, little is known about how this content engages us over time. Here, we show that narrative information alone synchronises heart rate across viewers, reflecting a shared state of engagement; and low-level audio-visual saliency had little effect on viewer physiology when viewing content with a strong narrative. Synchrony appears to increase during moments of greater narrative importance, for example when a twist in the plot is revealed.

### Data availability

Data and code used to reproduce all analyses in this paper are available at https://osf.io/ghtbw/. All participants provided informed consent for anonymised data to be made available online. Pre-registration of this study is available at the same location.
